# Health empowerment and access to health coverage among immigrants in France: the Makasi intervention

**DOI:** 10.1093/eurpub/ckac129.457

**Published:** 2022-10-25

**Authors:** M Bousmah, A Gosselin, K Coulibaly, A Ravalihasy, A Desgrées du Loû

**Affiliations:** 1 Ceped, Université Paris Cité, IRD, INSERM, Paris, France; 2 CNRS, French Collaborative Institute on Migrations, Aubervilliers, France; 3 Ined, National Institute for Demographic Studies, Aubervilliers, France

## Abstract

**Background:**

The Makasi community-based research project offered an innovative health-related empowerment intervention to immigrants from sub-Saharan Africa living in precarious situations in the greater Paris area, to reduce their social vulnerability and their exposure to HIV. Our objective is to evaluate the impact of the intervention on access to health coverage in this population.

**Methods:**

Participants were recruited based on precariousness criteria in public places in Ile-de-France (squares, railway stations, markets, etc.) by mobile teams of social workers and health mediators. Following a stepped-wedge design, participants were randomised into two groups receiving the intervention sequentially (immediately in one group and 3 months later in the other). We evaluated the impact of the Makasi intervention on access to health coverage among 821 individuals observed at 0, 3, and 6 months, between 2018 and 2021. We implemented random-effects panel models - allowing for unobserved heterogeneity - using a Heckman selection approach to correct for attrition. Finally, we used seemingly unrelated regressions (SUR) to examine the extent to which the effect of the intervention was mediated by health-related empowerment.

**Results:**

Participants - 77% of which were men - had been living in France for 4 years on average. 44% of them had no health coverage at the time of inclusion. Our results provided evidence for a significant impact of the Makasi intervention on participants’ access to health coverage, with an 18 percentage-point increase in the probability of accessing health coverage 6 months after having received the intervention (p < 0.01). The mediation analysis revealed that this effect operated partly through an empowerment process in terms of knowledge of social and health resources.

**Conclusions:**

We showed that a health empowerment intervention provided by social workers and health mediators largely favours access to health rights for immigrants in precarious situations.

**Key messages:**

• A health empowerment intervention improved access to health coverage among immigrants from sub-Saharan Africa living in precarious situations in France.

• Improvement in access to health coverage was found to be partly mediated by reinforcement of participants’ health literacy in terms of social and health resources.

